# Ultrasensitive SERS detection *via* hydrodynamic focusing coupled with electric field induced migration

**DOI:** 10.1039/d6an00722h

**Published:** 2026-09-28

**Authors:** Monika Poonia, Zachary D. Schultz

**Affiliations:** a Department of Chemistry and Biochemistry, The Ohio State University Columbus OH 43210 USA schultz.133@osu.edu

## Abstract

Surface-enhanced Raman spectroscopy (SERS) offers a powerful platform for ultrasensitive molecular detection, yet its performance is often limited by inefficient analyte transport to plasmonic hotspots. In this work, we present a hybrid microfluidic-SERS detection platform that integrates hydrodynamic focusing and electric field-induced migration to overcome diffusion limitations and enhance molecular delivery to the sensing interface. The system employs a sheath-flow microfluidic device with a thermally evaporated silver nanostructured substrate, serving both as a SERS-active surface and as an electrochemical working electrode. Rhodamine 6G, a model organic dye, was used to evaluate performance under four flow and biasing conditions. The strongest SERS signals were achieved under combined sheath flow and −1 V electric field, demonstrating a synergistic enhancement from both physical and electrokinetic focusing mechanisms. Quantitative analysis yielded Langmuir adsorption behavior over the tested range of concentration (0.1 nM–10 μM) and a limit of detection of 0.076 nM. Mechanistic confirmation was obtained through Stark-shift measurements of 4-mercaptobenzonitrile, which exhibited a measurable blue shift in the C≡N vibrational mode under +5 V bias, verifying the presence of localized electric fields at the metal–solution interface. This dual-mode transport-enhancement strategy provides a robust and generalizable method for improving sensitivity and control in microfluidic SERS detection platforms, with broad potential applications in biomedical diagnostics, environmental monitoring, and trace contaminant analysis.

## Abstract 2

An applied electric field to a SERS substrate is shown to improve detection of analyte molecules in a flow cell.

## Graphical abstract

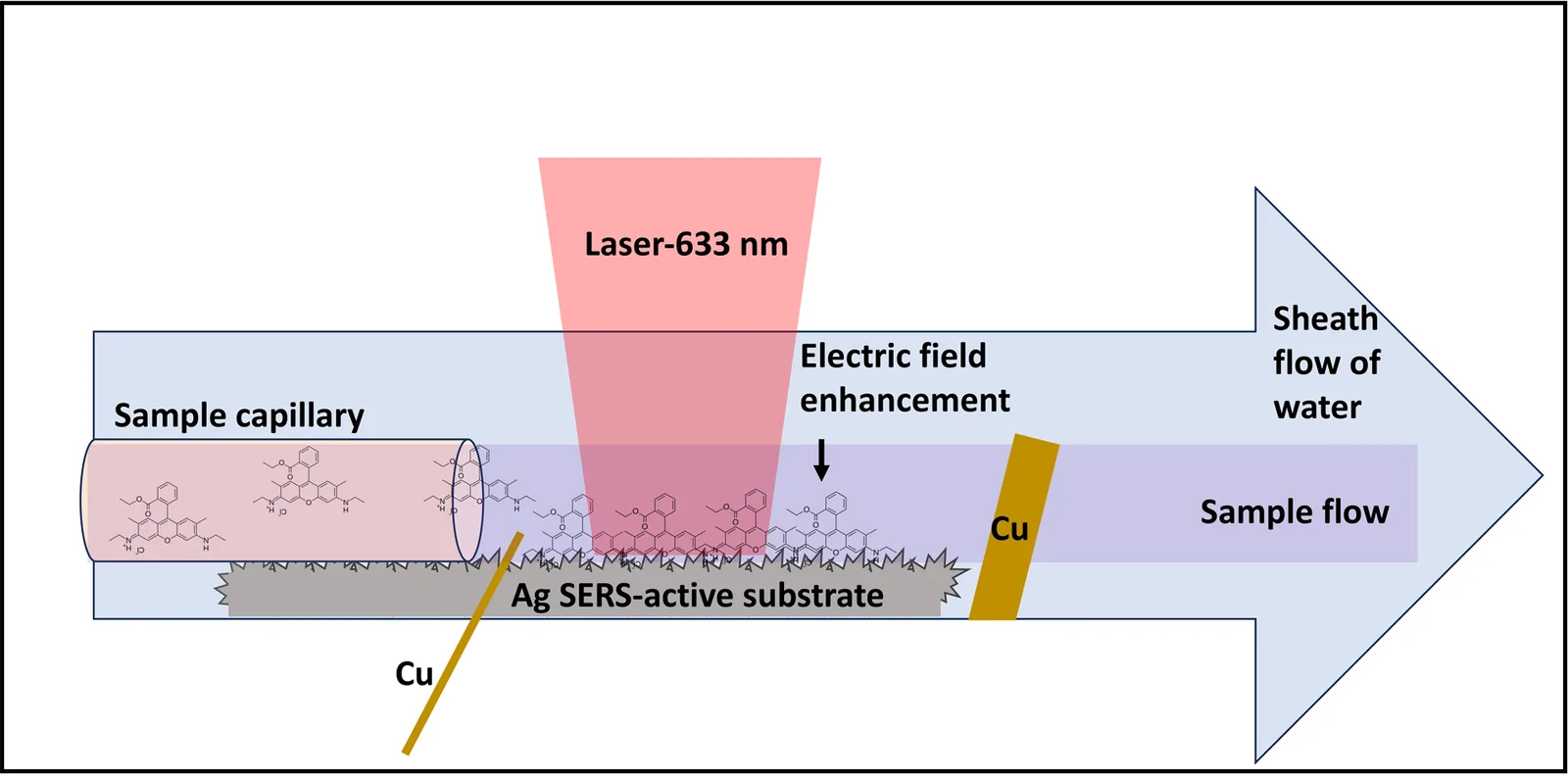

## 1. Introduction

Sensitive detection of trace-level analytes is of critical importance in diverse fields such as environmental monitoring, food safety, and biomedical diagnostics. The ability to identify and quantify minute concentrations of chemical or biological species enables early detection of contaminants, and hazardous compounds, thereby supporting timely decision-making and risk mitigation. Among the techniques available, surface-enhanced Raman spectroscopy (SERS) has gained prominence due to its capability for ultrasensitive and label-free molecular detection with fingerprint specificity.^1–5^ The enhancement in SERS arises primarily from the localized surface plasmon resonance (LSPR) effect of noble metal nanostructures, which amplifies Raman signals of molecules situated within plasmonic “hotspots” by several orders of magnitude.^6–8^ In addition to electromagnetic enhancement, chemical enhancement mechanisms involving charge transfer between the analyte and metal surface can further contribute to signal amplification. Despite its inherent sensitivity, practical deployment of SERS is often constrained by challenges in reliably delivering analyte molecules into the nanoscale enhancement regions. Over time, SERS has evolved through integration with various complementary techniques aimed at improving its accuracy, sensitivity, and reproducibility.^9^ In fluidic systems, analyte adsorption onto randomly distributed hotspots is largely diffusion-limited, resulting in suboptimal signal reproducibility and low detection efficiency, especially at very low analyte concentrations. This limitation is particularly critical for applications involving rapid screening or continuous sensing of low-abundance molecular species.^10,11^

To address these challenges, recent research has explored the integration of microfluidics with SERS-active substrates.^11–14^ Microfluidic platforms provide precise control over fluid flow and sample handling, making them well suited for point-of-use and in-line sensing applications. Notably, hydrodynamic focusing, wherein sheath flows confine the analyte stream into a narrow central flow path, has emerged as an effective strategy to spatially direct analyte toward the substrate surface, thereby increasing the probability of interaction with plasmonic hot spots.^15,16^ Several studies have demonstrated enhanced SERS signal intensity and reproducibility using sheath-flow configurations, as these systems effectively minimize analyte dispersion and optimize detection zone residence time.^17^ In parallel, electrochemical SERS (EC-SERS) has opened new avenues for enhancing analyte–substrate interaction and tuning the local electronic environment at the surface.^18–20^ Applying an external electric field can facilitate the electrophoretic migration of charged analytes, promote molecular orientation favorable for Raman scattering, and in some cases dynamically tune the plasmon resonance of the metal nanostructures.^21,22^ These effects collectively offer another dimension of control to improve SERS signal strength, especially for low-abundance and weakly interacting analytes. Despite these promising advancements, there remains a gap in the synergistic integration of both hydrodynamic confinement and electric field modulation in a unified sensing platform. Most existing microfluidic SERS systems either focus on passive confinement or rely solely on static substrate enhancements. Limited work has addressed how externally applied fields might dynamically modulate both analyte transport and plasmonic behavior in real-time within a microfluidic environment. Bridging this gap could significantly enhance the sensitivity, selectivity, and responsiveness of SERS-based detection platforms.

Although hydrodynamic confinement and electrochemical control have each been explored for SERS measurements, their combination provides an opportunity to simultaneously control analyte transport and interaction with the SERS-active surface. In this work, hydrodynamic confinement is coupled with electric-field-induced analyte migration to actively direct analytes toward an Ag substrate. Notably, this approach operates without a supporting electrolyte while maintaining measurable SERS enhancement and an electrochemical response. Using Rhodamine 6G as a model compound, we demonstrate that the synergistic effect of hydrodynamic and electrokinetic focusing markedly enhances detection sensitivity, enabling reliable SERS detection at sub-nanomolar concentrations. To further elucidate the underlying mechanisms, we employed 4-mercaptobenzonitrile as a probe molecule and performed cyclic voltammetry under similar experimental conditions, which produced a measurable current response. Stark-shift analysis further demonstrated a measurable blue shift in the C≡N vibrational mode under an applied bias of +5 V. This shift provides direct evidence of localized electric fields at the metal–solution interface, confirming the active role of electrochemical modulation in the system. This electrolyte-free configuration eliminates the need to introduce an additional supporting electrolyte into the sample stream, which may benefit applications where electrolyte composition could interfere with analyte chemistry or subsequent analysis. Thus, the key advance of this platform is the integration of hydrodynamic and electrical control of analyte transport in an electrolyte-free SERS configuration, rather than either transport mechanism alone. This approach not only addresses key limitations in analyte delivery and signal enhancement, but also provides a versatile platform for real-time, low-volume molecular sensing with potential applications in environmental and clinical diagnostics.

## 2. Materials and methods

### 2.1 Materials and chemicals

Rhodamine 6G (R6G), 4-mercaptobenzonitrile (4-MBN), and sodium hydroxide pellets (≥97%, ACS Reagent) were purchased from Sigma-Aldrich (St Louis, MO) and used without further purification. Stock solutions of R6G were prepared in ultrapure deionized water (18.2 MΩ cm, Thermo-Scientific Genpure System) at a concentration of 1 mM and diluted to desired working concentrations (10 μM to 1 nM) prior to use. All solutions were prepared fresh before each experiment and stored in the dark to avoid photodegradation. Bare fused silica capillary with 150 µm outer diameter and 75 µm inner diameter was purchased from Polymicro Technologies (Phoenix, AZ).

### 2.2 SERS substrate fabrication

Thermally evaporated SERS substrates were prepared as previously described.^23^ In brief, silver pellets (Kurt J. Lesker Company, Jefferson Hills, PA) were thermally evaporated onto commercially available anodized aluminum oxide (AAO) filters (Anodisc 13 mm, Whatman) with 0.2 µm pore size with a film thickness between 700 and 1100 nm. The resulting substrate was immersed in 0.1 M NaOH for 4 hours to dissolve the AAO. Once fully removed, the resulting metal films were rinsed thoroughly with ultrapure deionized water.

### 2.3 Microfluidic device design and fabrication

The microfluidic sheath-flow device configuration is previously described the Schultz lab.^12,24^ In brief, the flow cell consists of a fluorinated ethylene propylene (FEP) base plate equipped with inlet and outlet ports to enable sheath flow. A silicone gasket is placed on the base plate, followed by a glass slide pre-drilled to align with the flow ports. The prepared substrate is positioned on the slide and secured using clear nail polish (LA Colors, Ontario, CA). A capillary with a 75 µm inner diameter, with outer coating removed over the first ∼10 mm, is directly attached to the SERS substrate to position the focused stream over the SERS-active region. A 2 mm wide flow channel is defined using a 500 µm thick silicone gasket. A cover slip is then placed over the gasket, and the assembly is sealed by screwing down a stainless steel top plate. The sample solution was introduced at a flow rate of 1 µL min^−1^, while the sheath solution was delivered at 30 µL min^−1^, giving a sample-to-sheath flow-rate ratio of 1 : 30. Both the sample and sheath liquid flow rates were regulated using a syringe pump (Model NE-500 OEM, New Era Pump Systems Inc., Farmingdale, NY).

### 2.4 SERS instrumentation and acquisition parameters

Raman spectra were collected using a home-built Raman spectrometer equipped with a 633 nm He–Ne laser. A 40×, NA = 0.8, water immersion objective from Olympus was used to focus the excitation laser onto the detection region through the transparent microfluidic channel. The laser power at the sample was maintained at ∼1 mW to avoid thermal effects or photobleaching of the analyte. Raman spectra were acquired sequentially, each with a 250 ms acquisition time. The scattered light was directed into an Andor Shamrock 303i spectrograph and detected with an Andor iDus 401 CCD. Each SERS spectrum was acquired with an integration time of 250 ms, and 500 consecutive scans were averaged to improve signal-to-noise ratio.

### 2.5 Electrochemical measurements

Electrochemical measurements were performed using a CH Instruments Model 660D Potentiostat. The Ag substrate functioned as the working electrode, with electrical contact made *via* a copper wire connected to its edge. A strip of copper tape placed adjacent to the substrate acted as the auxiliary electrode. The working and auxiliary electrodes were separated by approximately 2 mm. No visible discoloration, gas-bubble formation, or substantial electrode degradation was observed during the experiments using the Cu tape counter electrode. Nevertheless, low-level Cu dissolution or potential interfacial effects cannot be excluded, as these were not directly quantified. A flow-through reference probe (Microelectrodes, Inc.) connected to the outlet serves as an Ag/AgCl electrode. All potentials reported in this study are referenced to the Ag/AgCl electrode. In the present electrode configuration, no additional supporting electrolyte was added to the sample stream. Because the sensing platform operates without an added supporting electrolyte, the applied voltage represents the applied cell bias and should not be interpreted as the exact potential drop across the SERS interface. Fig. 1 illustrates the sheath flow cell with its electrochemical configuration, along with a photograph of the sheath flow cell setup showing the electrical contacts. Four distinct experimental configurations were employed to systematically investigate the individual and combined effects of hydrodynamic focusing and electric field-driven analyte migration. In the first configuration, the analyte (R6G) was introduced over the Ag SERS substrate without any sheath fluid or applied external potential, providing a baseline for assessing passive transport and SERS signal intensity. The second setup involved hydrodynamic focusing of the analyte onto the SERS substrate, again without applying an external potential, to isolate the influence of hydrodynamic focusing alone. In the third configuration, a potential of −1 V was applied to the Ag substrate without hydrodynamic focusing, thereby promoting electrophoretic migration of the cationic R6G molecules toward the negatively charged plasmonic surface. The fourth and final configuration combined hydrodynamic focusing with an applied electric bias throughout the measurement period, enabling real-time analyte accumulation and enhanced molecular interactions within the SERS hotspots. For all experiments, flow rates and laser excitation conditions were kept constant to ensure that SERS intensity comparisons were directly attributable to the experimental variables under investigation.

**Fig. 1:**
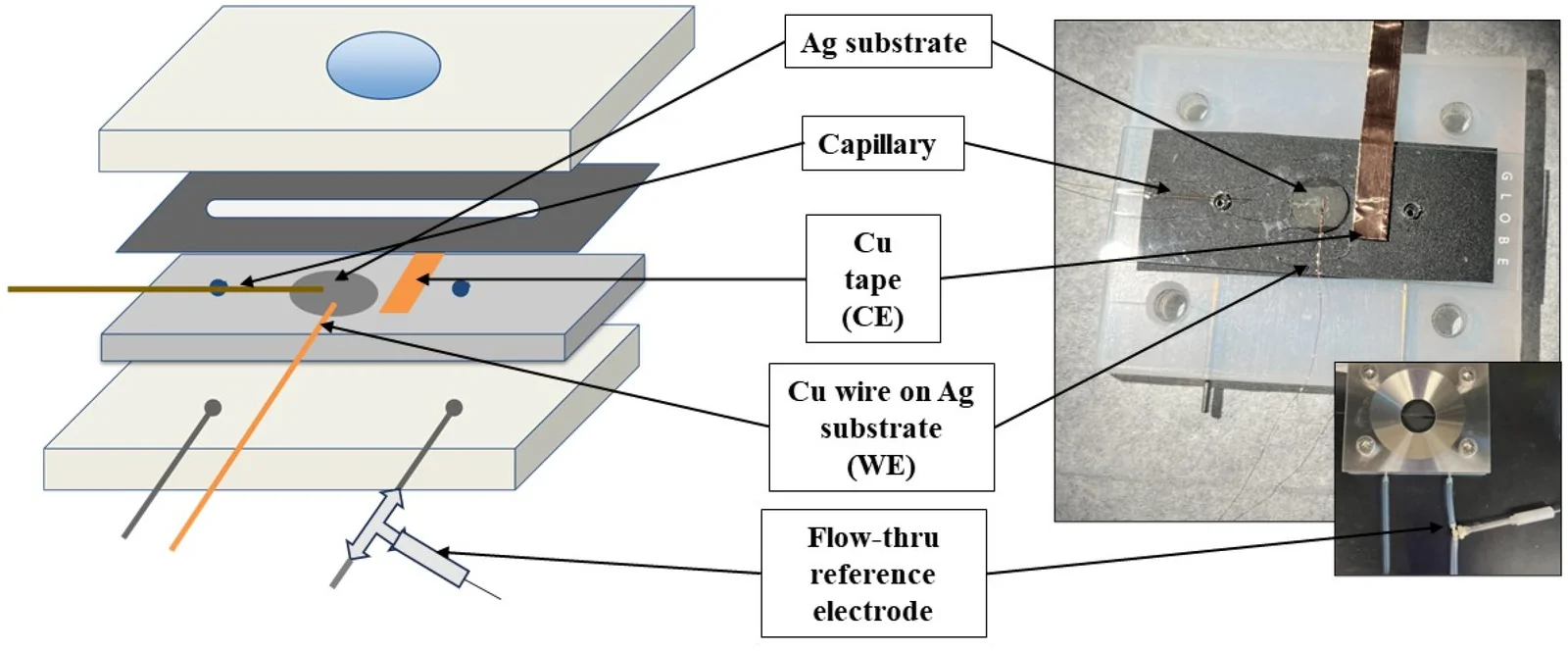
Microfluidic platform configured for hydrodynamic focusing and electrochemical SERS measurements. Fluidic channels in the Teflon baseplate provide the sheath flow inlet and outlet. A 75 µm inner diameter capillary delivers the analyte into the larger flow channel over the SERS substrate. Electrochemical control is enabled by a Cu wire that makes the SERS substrate the working electrode (WE) while copper tape in the flow channel serves as the counter electrode (CE). A flow-through reference probe attached to the outlet functions as an Ag/AgCl electrode. The inset photo depicts the assembled flow cell.

For each configuration, 500 consecutive spectra were collected from a single substrate region to assess temporal spectral variation, not independent experimental reproducibility. Accordingly, the reported variability does not capture variation among substrates, fabrication batches, or experimental days. Future studies should include independent measurements across these factors to quantify substrate heterogeneity and calibration uncertainty performance.

### 2.6 Data processing and analysis

Raw SERS spectra were exported and processed using MATLAB (R2023a, MathWorks). Baseline correction was performed using rolling circle fitting algorithm with a radius of 10 000.^25^ Peak intensities were quantified at key vibrational modes of R6G. Data were plotted as mean ± standard deviation (*n* = 500), and enhancement trends were evaluated across all four experimental conditions. MATLAB was also used to perform peak-fit analysis. Results were plotted using Origin plotting tool (OriginPro 9).

## 3. Results and discussion

The performance of the integrated microfluidic SERS setup was evaluated under four distinct experimental conditions to understand the individual and combined effects of hydrodynamic focusing and electric field-induced analyte migration. These conditions included: (A) the absence of both sheath flow and applied potential (0 V, sheath flow off), serving as a diffusion-limited control; (B) the use of sheath flow to confine the analyte stream without any applied potential (0 V, sheath flow on); (C) the application of an electric bias of −1 V with no sheath flow (−1 V, sheath flow off); and (D) the simultaneous application of sheath flow and a −1 V bias across the detection region (−1 V, sheath flow on). Average SERS spectra of R6G at different concentrations measured under these conditions are shown in Fig. 2A–D. Fig. S1 in SI presents the average raw SERS spectra of R6G at various concentrations under the same conditions.

**Fig. 2:**
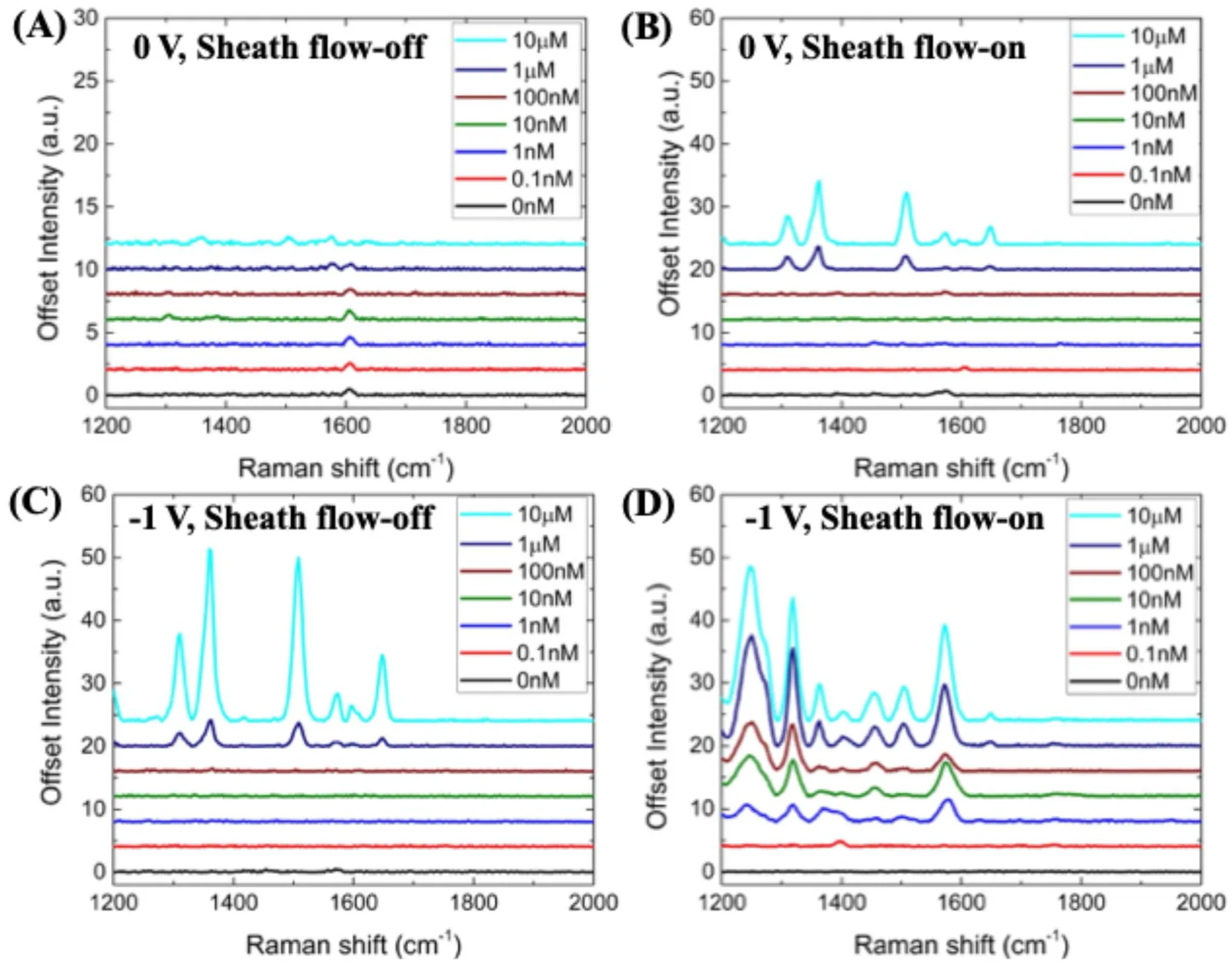
Average SERS spectra of R6G at different concentrations, measured under four distinct conditions: (A) diffusion-limited (0 V, sheath flow off), (B) sheath-flow only (0 V, sheath flow on), (C) electric field only (−1 V, sheath flow off), and (D) combined sheath flow and electric field (−1 V, sheath flow on). The SERS spectra are vertically offset for clarity.

The SERS spectra of R6G exhibited prominent peaks at 1312, 1360, 1505, 1574, and 1648 cm^−1^. The 1312 cm^−1^ band corresponds to a coupled vibration involving the hybrid xanthene/phenyl rings and the ethylamino group, 1360 cm^−1^ to a symmetric xanthene ring C–C stretch, 1505 cm^−1^ to xanthene C–C stretching, 1574 cm^−1^ to phenyl ring C–C stretching, and 1648 cm^−1^ to the high-frequency xanthene C=C stretch.^26–28^ Under the diffusion-limited condition (0 V, sheath flow off), only weak SERS signal intensities were detected, even at the highest tested R6G concentration of 10 μM (Fig. 2A). This limited response is primarily attributed to inefficient mass transport, as analyte molecules rely solely on random diffusion to reach the plasmonic hotspots. Without the assistance of flow or an applied electric field, the movement of R6G molecules toward the active sensing regions is significantly restricted, resulting in reduced interaction with the substrate and consequently lower signal intensities. A moderate improvement was observed under the sheath-flow-only condition (0 V, sheath flow on), where the analyte stream was hydrodynamically confined over the detection region, increasing the probability of interaction with the plasmonic substrate. At the higher concentrations of 1 µM, all five R6G peaks exhibit low intensities with a modest enhancement detected at 10 µM (Fig. 2B).

When only the electric field was applied (−1 V, sheath flow off), a further enhancement in spectral intensity was observed (Fig. 2C), attributable to electrophoretic migration of the cationic R6G molecules toward the negatively biased Ag substrate. This supports the hypothesis that external electric fields can enhance analyte–substrate interaction by facilitating directed transport.^19,29^ The calibration curves for conditions (A) 0 V, sheath flow off, (B) 0 V, sheath flow on and (C) −1 V, sheath flow off are shown in SI Fig. S2. The slope of the linear fit was used to calculate the limit of detection (LOD), applying the IUPAC convention:LOD = 3*σ*/*S* where *σ* is the standard deviation of the spectral noise from the blank solution, and *S* is the slope of the linear fit of the calibration curve. The LOD was found to be approximately 10.04 µM, 2.1 µM and 729 nM for conditions (A) 0 V, sheath flow off, (B) 0 V, sheath flow on and (C) −1 V, sheath flow off, respectively.

The highest SERS signal intensity was consistently achieved under the combined condition of sheath flow and electric field (−1 V, sheath flow on) (Fig. 2D). This condition yielded the strongest spectral features, indicating a synergistic effect wherein hydrodynamic focusing enables spatial control of the analyte stream, while the applied bias promotes molecular accumulation at the active detection surface.

All primary peaks remained stable except for the 1312 cm^−1^ band, which shifted to 1318 cm^−1^ only when both sheath flow and −1 V were applied.^30^ Interestingly, under this same condition, an additional peak appeared at 1248 cm^−1^. This peak was not observed under any of the other three conditions, indicating that it is activated specifically by the combined effects of applied potential and dynamic flow. The emergence of the band at 1248 cm^−1^ and the shift of the band from 1312 to 1318 cm^−1^ under combined sheath flow and applied potential may reflect changes in R6G adsorption, molecular orientation, or the local electronic environment at the Ag surface. However, these spectral changes may also involve concentration-dependent adsorption, aggregation, local pH variations or interfacial electrochemical processes. Cyclic voltammetry of 10 mM MBN under electrolyte-free conditions showed measurable current responses and differences between the forward and reverse scans (Fig. S3), indicating that electrochemical effects cannot be completely excluded. Potential-dependent changes in SERS spectra can arise from changes in molecular adsorption or orientation at metal surfaces, including for R6G.^21^ At negative potentials, hydrogen evolution and associated local pH changes near the electrode may further contribute to the observed response.^30^ Thus, the observed spectral changes under −1 V and flow likely arise from a combination of electrically controlled analyte transport, changes in adsorption or molecular orientation, and possible interfacial electrochemical effects. Because the present platform operates without an added supporting electrolyte, the CV response may not be directly comparable to that of conventional electrolyte-supported electrochemical systems.

To quantitatively assess the sensitivity of the system under optimized conditions (−1 V, sheath flow on), a calibration series was performed using R6G concentrations ranging from 0.1 nM to 10 μM. The characteristic Raman peak at 1318 cm^−1^ was selected for intensity-based quantification due to the strong response across the range of concentrations.

Fig. 3 represents the resulting calibration curve, showing peak intensity at 1318 cm^−1^ as a function of analyte concentration. Langmuir–Freundlich adsorption behavior was observed between 0.1 nM and 10 μM (*R*^2^ = 0.98), with signal saturation occurring beyond 1 μM. The LOD was estimated from the blank response and the variability of the blank measurements. The LOD response, *I*_LOD_, was calculated as:*I*_LOD_ = *I*_blank_ + 3*σ*_blank_where *I*_blank_ is the mean intensity of the blank measurements and *σ*_blank_ is standard deviation of the blank measurements.^31^

**Fig. 3:**
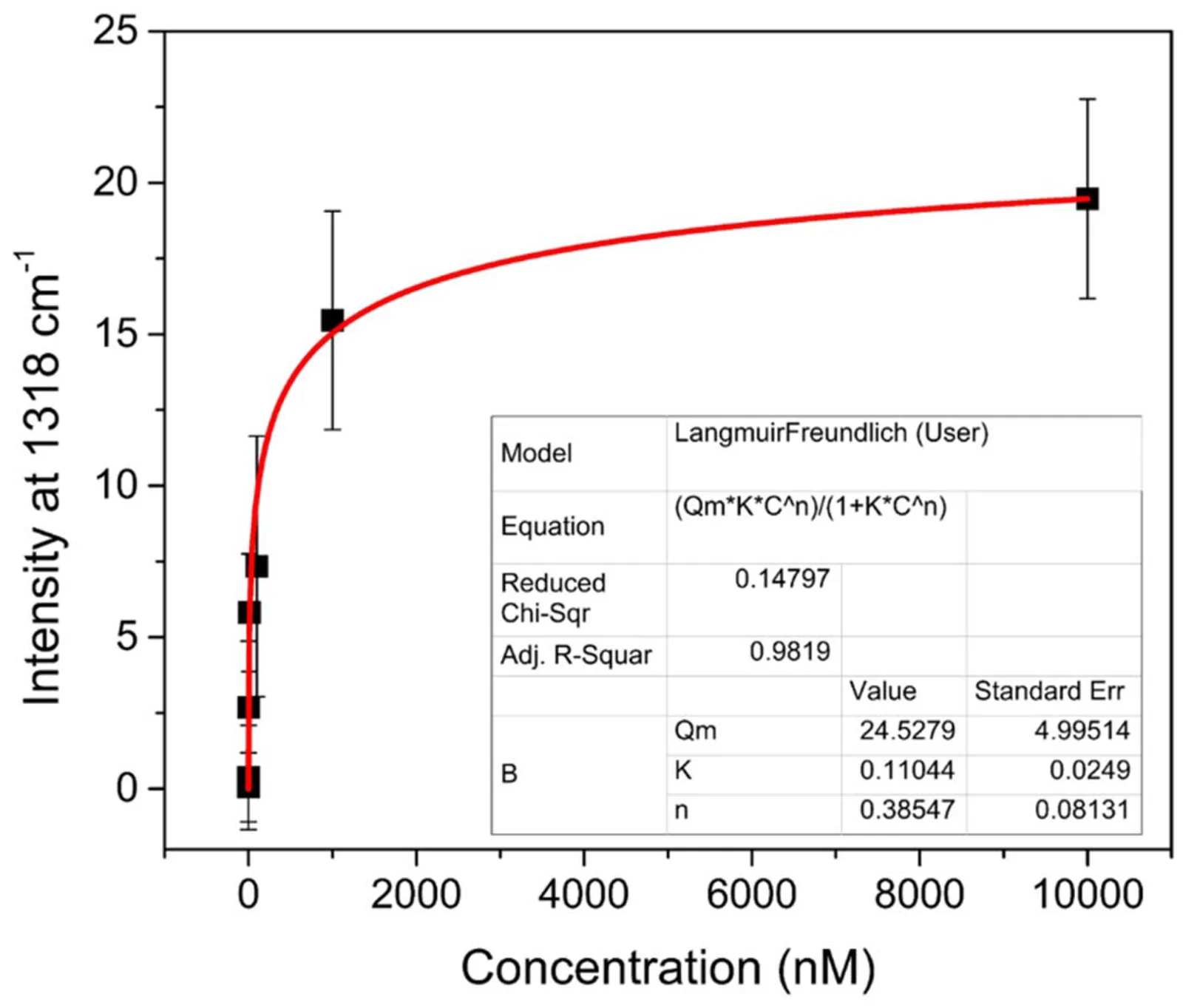
Calibration curve for R6G measured under optimized SERS conditions (−1 V bias, sheath flow on). Data points represent the mean peak intensity at 1318 cm^−1^ ± standard deviation (*n* = 500). The red line indicates the fitted regression, demonstrating Langmuir–Freundlich adsorption behavior across the 0.1 nM to 10 μM concentration range (*R*^2^ = 0.98).

Following procedures described in the literature,^32,33^ the calculated intensity can be equated to a concentration *C*, through rearrangement of the Langmuir–Freundlich isotherm as shown in equation:
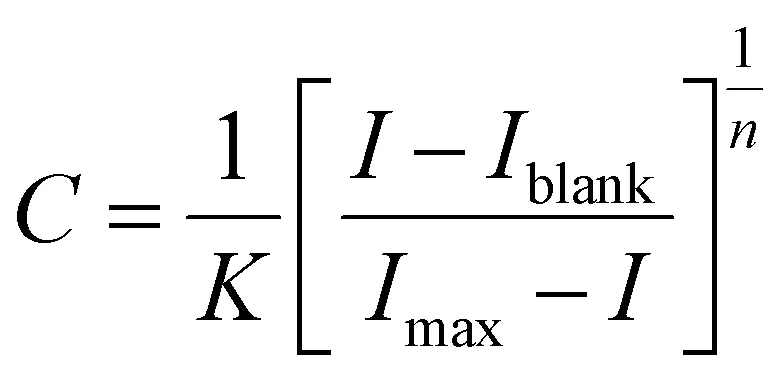


A low detection limit of 0.076 nM was determined for R6G, indicating high sensitivity suitable for trace-level detection. The low LOD clearly indicates that both hydrodynamic focusing and electric field migration play complementary roles in improving SERS sensitivity. In the absence of these mechanisms, detection relies on passive diffusion, which limits reproducibility and signal intensity due to random molecular distribution. The use of sheath flow introduces control over analyte positioning by focusing the sample stream into a narrow laminar flow aligned with the substrate surface. This increases interaction frequency with plasmonic hotspots while reducing sample dispersion. In parallel, application of a −1 V potential enhances the delivery of positively charged R6G molecules toward the negatively charged Ag electrode, increasing the local analyte concentration at the SERS-active region. The combination of these two strategies results in improved signal and a significantly lower detection limit compared to either approach alone. These findings are in strong agreement with prior studies on hydrodynamic SERS^14,17^ and electrochemical SERS enhancement,^34–37^ but the integration of both mechanisms into a single detection platform represents a notable advancement. The tunable nature of the electric field also provides an additional parameter for optimizing detection in complex samples, especially for analytes with varying charge states or mobilities. A comparison of the present approach with representative hydrodynamic/sheath-flow SERS and electrochemical SERS platforms, including their analytes, detection limits, electrolyte requirements, and approaches for analyte transport and detection, is provided in Table S1 in SI.

To quantify the signal differences among the four experimental conditions, we considered relative Raman signal enhancement based on the measured intensity at the selected R6G characteristic peak. Relative to condition (A) 0 V, sheath flow off, the observed signal increased by 10.83-, 28.27-, and 85.14-fold for conditions (B) 0 V, sheath flow on, (C), −1 V, sheath flow off and (D) −1 V, sheath flow on, respectively. Because a conventional Raman reference spectrum of R6G under matched conditions was not available, we refer to these values as relative signal enhancement rather than conventional SERS enhancement factors. The values are intended to quantify the experimentally observed signal increase between the four conditions and should not be interpreted as absolute SERS enhancement factors.

### 3.1 Electric field-induced Stark shift

To further investigate the role of applied electric field in modulating molecular interactions at the plasmonic surface, additional SERS experiments were conducted using 4-mercaptobenzonitrile (4-MBN), a well-known Stark probe that chemisorbs onto noble metals through its thiol group while exposing the nitrile (C≡N) group toward the solution interface. MBN forms a stable self-assembled monolayer on silver substrates, and the C≡N stretch is highly sensitive to changes in interfacial electric field strength and direction. Thermally evaporated Ag substrates with a thickness of approximately 700 nm were functionalized with 4-MBN. The substrates were immersed in a 10 mM ethanolic solution of 4-MBN and incubated overnight to allow self-assembled monolayer formation. Following incubation, the substrates were soaked in NaOH solution for 4 h to dissolve the AAO filter paper backing. SERS spectra of 4-MBN were then recorded without sheath flow across the channel.

SERS spectra of 4-MBN were acquired at two applied potentials: 0 V and +5 V (*vs.* Ag/AgCl) under similar acquisition conditions. As shown in Fig. 4, the C≡N vibrational mode appeared at ∼2231 cm^−1^ at 0 V, consistent with reported values for 4-MBN monolayers on silver electrodes. Upon application of a +5 V bias, the C≡N stretching frequency exhibited a measurable blue shift of ∼3–4 cm^−1^, shifting between 2230–2234 cm^−1^. This shift is consistent with the vibrational Stark effect, wherein the energy of the C≡N dipole vibration can be modulated by the electric field at the interface. The shift suggests a nonzero but modest interfacial field present, and it is consistent with prior Stark-shift measurements on nitrile reporters.^38–40^ The absence of an added supporting electrolyte in the present system is expected to influence the interfacial electric field distribution and, consequently, the magnitude of the observed vibrational Stark shift.^41^ Under low ionic strength conditions, the electric field extends further into the solution phase with reduced screening, resulting in a more diffuse potential drop and a lower effective field strength at the probe molecule.^42^ This lower field strength reasonably accounts for the relatively modest shift observed for the C≡N vibrational mode. At the same time, the electrolyte-free configuration minimizes the addition of ion-specific interactions and supporting-electrolyte-related perturbations, providing a minimally modified environment for evaluating field-dependent molecular responses. Thus, while the observed Stark shift provides evidence of an electric-field effect within the device, its magnitude should not be interpreted as a quantitative measure of the interfacial electric field.

**Fig. 4:**
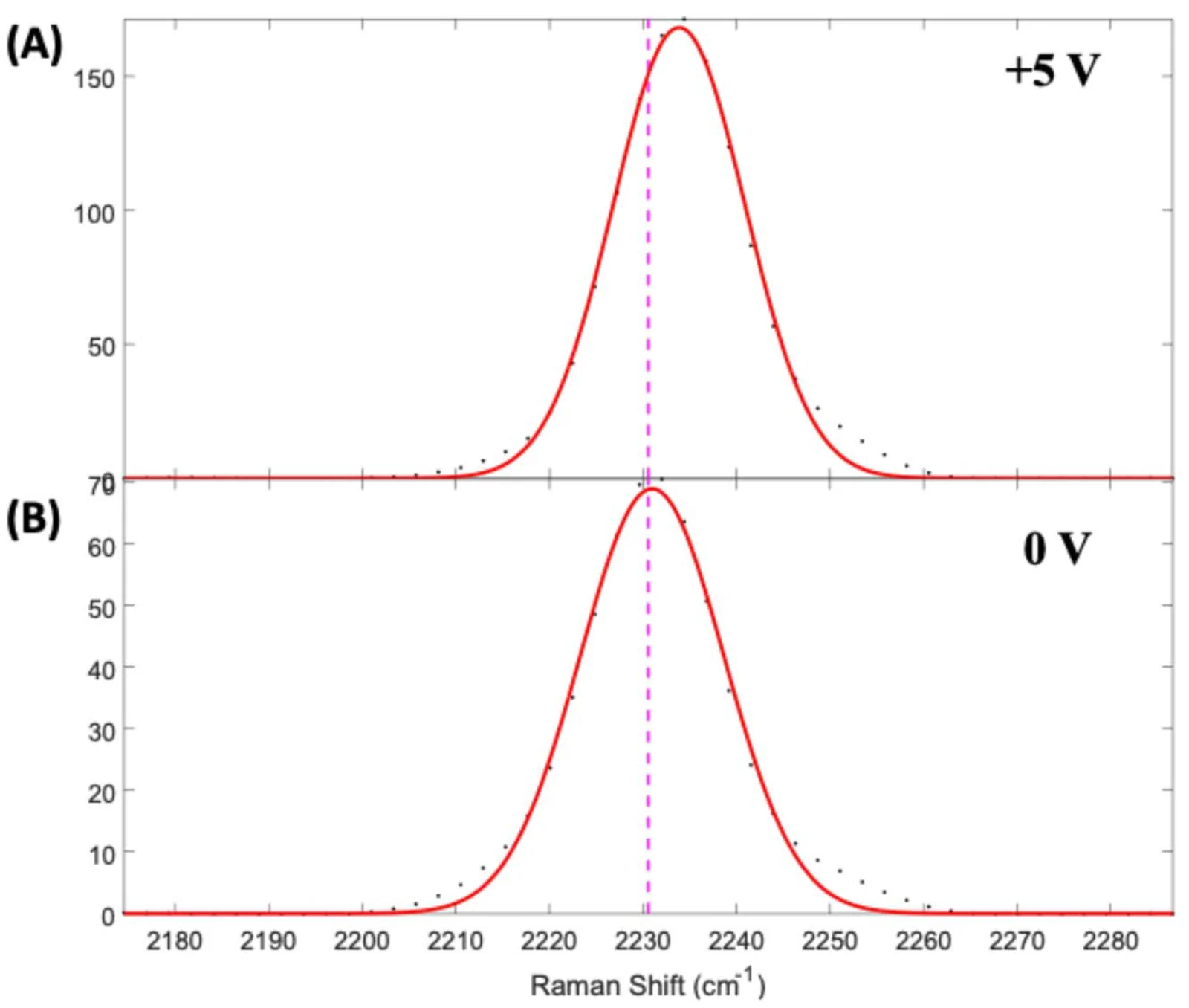
SERS spectra of 4-mercaptobenzonitrile (4-MBN) adsorbed on Ag substrate at (A) +5 V *vs.* Ag/AgCl and (B) 0 V. A Stark shift of ∼3–4 cm^−1^ is observed in the C≡N stretching band (from 2230.5 to 2234.4 cm^−1^) under positive bias, confirming electric field modulation at the interface.

It is important to note that the Stark measurement at +5 V using 4-MBN does not directly establish the mechanism responsible for the enhanced R6G response observed at −1 V. Rather, the Stark response supports the presence of an electric-field contribution within the device. The enhanced R6G response may additionally involve electrically influenced analyte transport, local concentration accumulation, changes in adsorption or molecular orientation, surface charging, and possible interfacial electrochemical effects. A systematic potential-dependent Stark study would be required to quantitatively establish the relationship between applied potential and the interfacial electric field.

Overall, the results obtained across the four tested conditions provide key insights into the role of fluidic and electrokinetic transport in enhancing SERS detection. Under static or bias-only conditions, diffusion-limited delivery restricted analyte accumulation at the silver surface, resulting in lower intensity and minimal spectral changes. Activation of sheath flow improved molecular focusing, bringing more analyte into the sensing region, however, without an electric bias, the delivery remained governed largely by hydrodynamic forces. The diffusion layer in the presence of hydrodynamic focusing still persists over several micrometers, requiring passive transport of the analyte to the SERS substrate. The most pronounced enhancement was observed when sheath flow was combined with a −1 V bias, suggesting a synergistic mechanism, convective focusing by sheath flow narrows the analyte stream and minimizes the diffusion layer, while the electric field actively drives cationic R6G toward the electrode surface. This dual focus increases surface coverage and hot-spot utilization, producing the highest SERS response. The improved detection observed here is consistent with lower limits of detection observed in capillary electrophoresis separations with SERS detection,^43^ relative to detection in the absence of electric fields.^12^ These results indicate that together hydrodynamic focusing and electric fields can not only increase analyte flux but may also modulate the molecular binding environment, adsorption or molecular orientation, and possible interfacial electrochemical effects contributing to improved spectral response and peak behavior.

## 4. Conclusions

This work demonstrates a hybrid microfluidic-SERS sensing strategy that leverages sheath flow and electrode bias to overcome mass transport limitations and achieve sensitive, controllable detection. By using R6G as a model analyte, the study demonstrates how hydrodynamic focusing from sheath flow significantly improves delivery of molecules to the sensing interface, while the applied bias further drives cationic species toward active hot spots, resulting in the highest SERS intensities observed among the tested conditions. Beyond signal enhancement, the applied field also produced distinct spectral changes, including field-induced vibrational shifts and the appearance of new peaks, suggesting altered molecular orientation or binding interactions at the silver electrode. Complementary experiments with 4-MBN further confirmed the presence of localized electric fields at the electrode surface, supporting the proposed mechanism of electrokinetic focusing combined with enhanced adsorption.

Quantitative analysis of the R6G response revealed behavior consistent with Langmuir–Freundlich adsorption, with a detection limit of approximately 0.076 nM, highlighting the sensitivity and reproducibility of the approach. Importantly, the observations from both R6G and 4-MBN not only validate the benefits of integrating hydrodynamic and electrokinetic mechanisms but also provide new mechanistic insights into how electric fields can modify molecular interactions and orientation in SERS systems. These results provide not only a mechanistic understanding of transport and field effects but also a blueprint for integrating multiple focusing strategies in next-generation SERS devices. Future work can extend this approach to multiplexed detection, real-time monitoring in complex matrices, and tailored electrode geometries to further optimize field distribution and signal gain for applications in diagnostics, environmental sensing, and trace analysis.

## Conflicts of interest

There are no conflicts of interest to declare.

## Supplementary Material

AN-151-D6AN00722H-s001

## Data Availability

The data supporting this article have been included as part of the supplementary information (SI). Supplementary information is available including: Figure S1, average SERS spectra; Figure S2, calibration curves under varios conditions; Figure S3, cyclic voltammogram wiht 10 mM MBN on the SERS substrate; and Table S1, reviewing other SERS flow platforms. See DOI: https://doi.org/10.1039/d6an00722h. Requests for data in a digital format (*e.g.* .csv) are available upon request.
