# Supplementary material for: Ultrasensitive SERS detection via hydrodynamic focusing coupled with electric field induced migration
Source: Analyst. 2026 Sep 28;151(21):6383–91. doi: 10.1039/d6an00722h (PMC13651769; doi:10.1039/d6an00722h)
Supplement: Supplementary file 1 [file AN-151-D6AN00722H-s001.pdf]

Supplementary Information (SI) for Analyst.
This journal is © The Royal Society of Chemistry

Ultrasensitive SERS Detection via Hydrodynamic Focusing Coupled with Electric Field Induced Migration

Monika Poonia[a], Zachary D. Schultz[a]*

[a]Department of Chemistry and Biochemistry, The Ohio State University, Columbus, OH 43210, USA

Corresponding author: schultz.133@osu.edu

List of Figures:

Figure S1: Average SERS spectra of Rhodamine 6G at various concentrations. (A) at 0 V, sheath flow off, (B) at 0 V, sheath flow on, (C) at -1 V, sheath flow off, and (D) at -1 V, sheath flow on.

Figure S2: Calibration curve for Rhodamine 6G measured under various conditions: (A) at 0 V, sheath flow off, (B) at 0 V, sheath flow on, (C) at -1 V, sheath flow off.

Figure S3: Cyclic voltammogram of 10 mM MBN recorded under electrolyte-free conditions over the investigated potential range.

List of Tables:

Table S1: Comparison of representative hydrodynamic/sheath-flow SERS and electrochemical SERS platforms with the present study, including analytes, detection limits, supporting electrolyte requirements, and key features of analyte transport and detection.

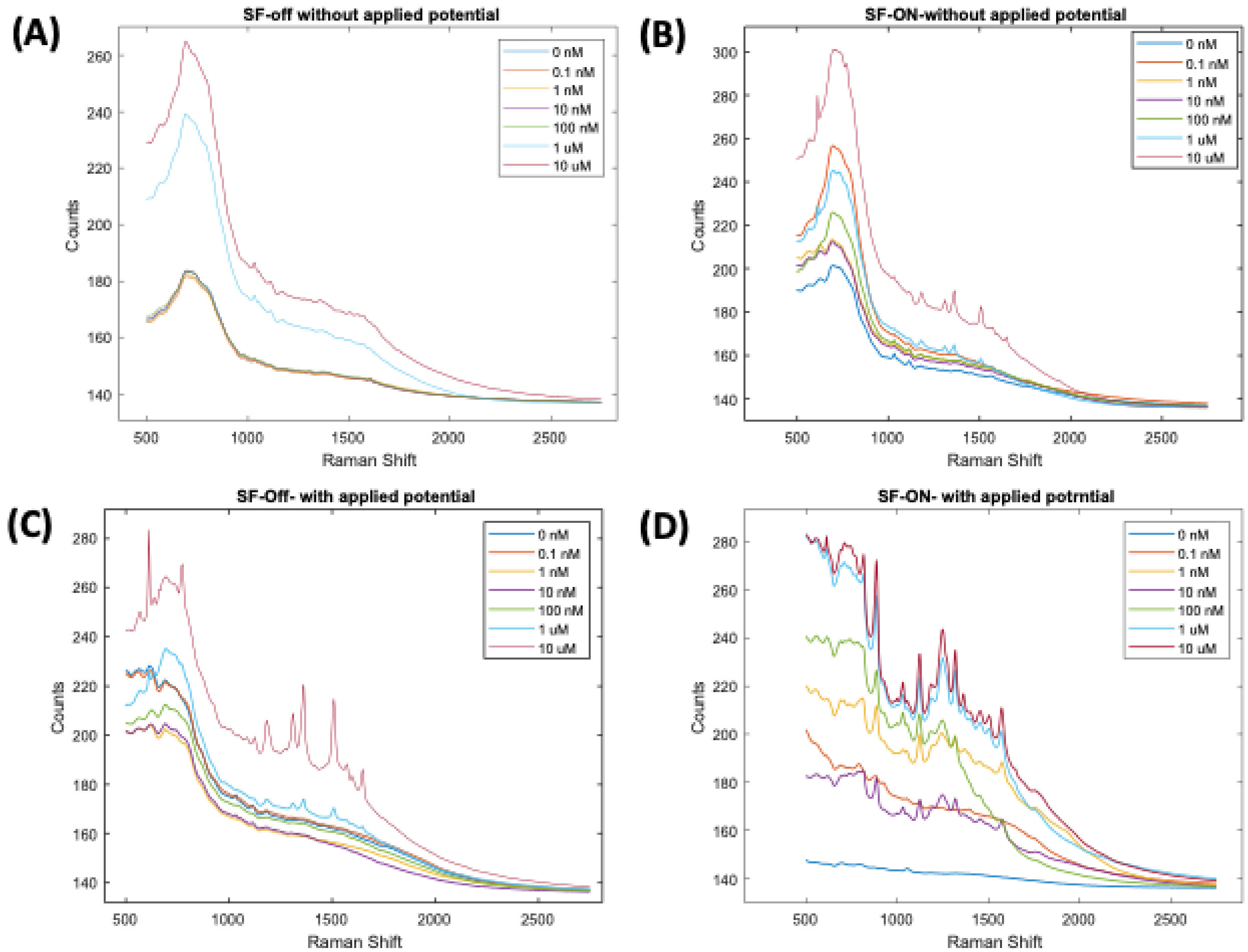

Figure S1: Average SERS spectra of Rhodamine 6G at various concentrations. (A) at 0 V, sheath flow off, (B) at 0 V, sheath flow on, (C) at -1 V, sheath flow off, and (D) at -1 V, sheath flow on.

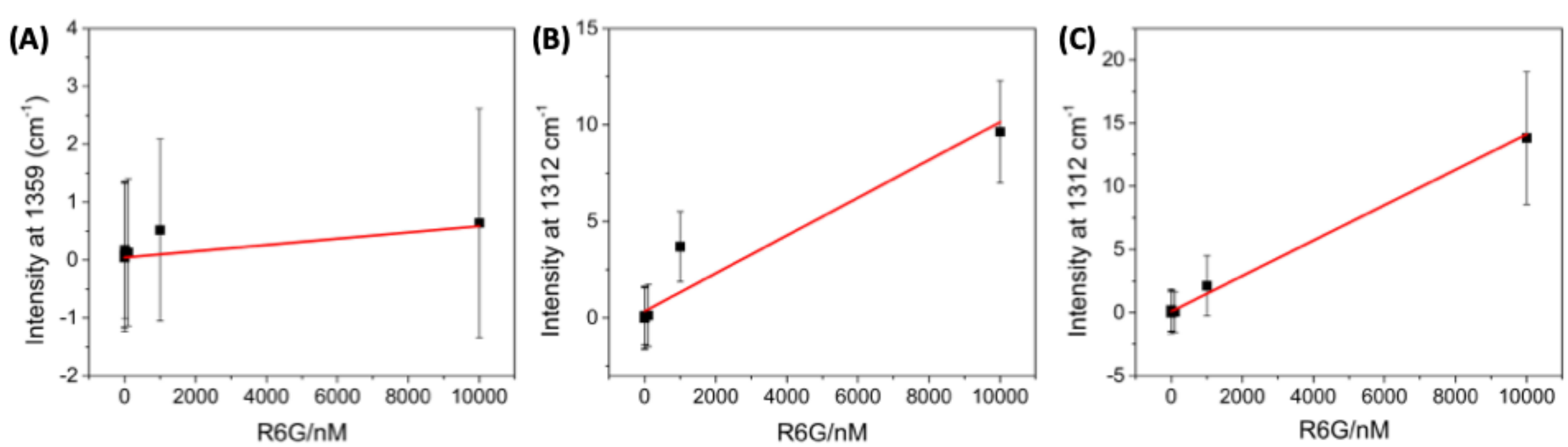

Figure S2: Calibration curve for Rhodamine 6G measured under various conditions: (A) at 0 V, sheath flow off, (B) at 0 V, sheath flow on, (C) at -1 V, sheath flow off.

Ref.	Approach	Flow / hydrodynamic confinement	Electrical / electrochemical control	Supporting electrolyte	Analyte(s)	Reported LOD	Key feature
(Negri et al., 2013)	Hydrodynamic-focusing SERS flow detector	Yes	No	No	R6G	1 nM	Sheath flow confines analyte near planar Ag SERS substrate; high-throughput SERS in flow
(Negri et al., 2014)	CZE–sheath-flow SERS	Yes	Electrophoretic separation	Yes, separation buffer	R6G, Rhodamine B, 5-TAMRA	nM-level detection	Combines electrophoretic transport/separation with sheath-flow SERS
(Negri & Schultz, 2014)	CZE–sheath-flow SERS	Yes	Electrophoretic separation	Yes, separation buffer	20 proteinogenic amino acids	10^{-5}–10^{-10} M	General-purpose online SERS detection following electrophoretic separation
(Bailey et al., 2015)	Sheath-flow SERS + electrochemical detection	Yes	Yes	Yes (0.1 M NaOH)	Riboflavin	1 nM SERS; 89–100 nM electrochemical	Simultaneous hydrodynamic focusing, SERS and amperometric detection
(Zhao et al., 2015)	EC-SERS	No	Yes	Yes (0.1 M NaF)	Uric acid	100 µM	Quantitative EC-SERS using multilayer Au/Ag substrate
(Moldovan et al., 2023)	EC-SERS	No	Yes	Yes	Thiabendazole	0.306 µM	Quantitative EC-SERS detection in apple juice using activated Au screen-printed electrodes

(Chotoye et al., 2023)	EC-SERS	No	Yes	Yes (0.1 M NaF)	Glutathione	5 μM	Direct EC-SERS detection in artificial saliva
(Huang & Hsiao, 2020)	Integrated microfluidic EC-SERS	Yes (microfluidic reservoir)	Yes	Yes	Uric acid	0.087 μM	Integrated three-electrode EC-SERS chip with nanostructured Au electrode
(Jones et al., 2024)	EC-SERS + ML	No	Yes	Yes (0.1 M NaF in measurements)	Uric acid, creatinine	0.127 and 0.354 μM	Potential-dependent quantitative multiplexed EC-SERS
**Present work**	**Hydrodynamic + electric-field-directed SERS**	**Yes**	**Yes**	**No supporting electrolyte**	**R6G**	**0.076 nM**	**Coupled hydrodynamic confinement and electric-field-induced migration in flow without supporting electrolyte**

Table S1: Comparison of representative hydrodynamic/sheath-flow SERS and electrochemical SERS platforms with the present study, including analytes, detection limits, supporting electrolyte requirements, and key features of analyte transport and detection.

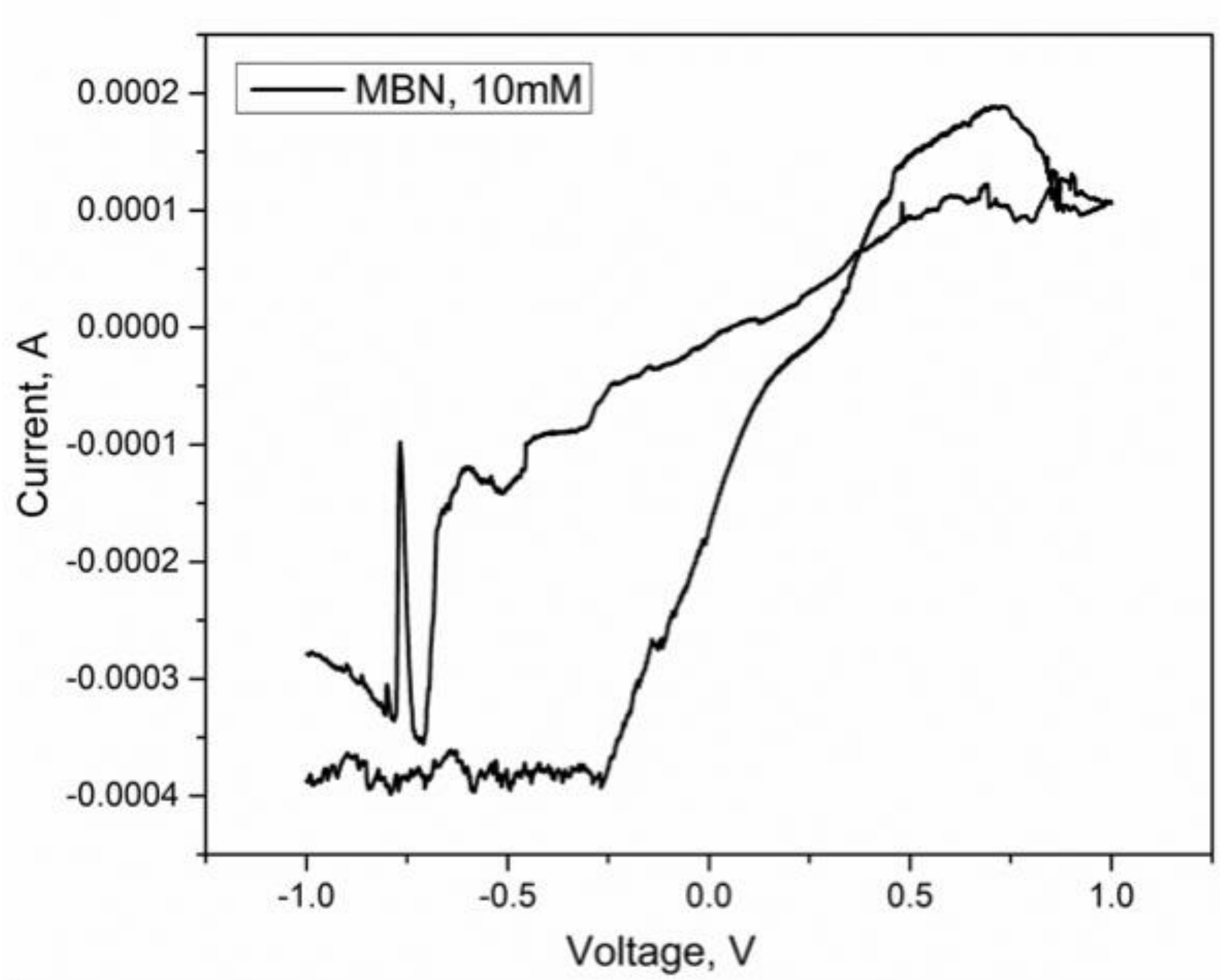

Figure S3: Cyclic voltammogram of 10 mM MBN recorded under electrolyte-free conditions over the investigated potential range.

Limit of detection:

LOD calculation for optimized conditions (−1 V, sheath flow on) from Langmuir-Freundlich isotherm behavior observed in calibration Figure 3:

The LOD was estimated from the blank response and the variability of the blank measurements. The LOD response , I_{LOD}, was calculated as:

$$I_{LOD} = I_{blank} + 3\sigma_{blank}$$

where I_{blank} is the mean intensity of the blank measurements and σ_{blank} is standard deviation of the blank measurements.

Concentration using the Langmuir–Freundlich model,

$$C = \frac{1}{K}\left[\frac{I - I_{blank}}{I_{max} - I}\right]^{1/n}$$

where (I) is the measured SERS intensity at concentration (C), (I_{blank}) is the baseline response parameter, (I_{max}) is the maximum response, (K) is the concentration-related fitting constant, and (n) is the Freundlich heterogeneity exponent.

Here, $I_{\mathrm{blank}} = 0.057$, $I_{\max} = 24.52$, $K = 0.11$, $n = 0.38$, and $I = 3.477$, from Figure 3.

$$C_{\mathrm{LOD}} = \frac{1}{0.11}\left[\frac{3.477 - 0.057}{24.52 - 3.477}\right]^{1/0.38}$$

$C_{LOD} \approx 0.076$ nM

References

Bailey, M. R., Pentecost, A. M., Selimovic, A., Martin, R. S., & Schultz, Z. D. (2015). Sheath-Flow Microfluidic Approach for Combined Surface Enhanced Raman Scattering and Electrochemical Detection. *Analytical Chemistry*, *87*(8), 4347–4355. https://doi.org/10.1021/acs.analchem.5b00075

Chotoye, S. A. B., Eisnor, M. M., Ball, R. B. E., & Brosseau, C. L. (2023). Development of an electrochemical surface-enhanced Raman spectroscopic biosensor for the direct detection of glutathione. *Journal of Raman Spectroscopy*, *54*(6), 587–595. https://doi.org/10.1002/jrs.6522

Huang, C.-Y., & Hsiao, H.-C. (2020). Integrated EC-SERS Chip with Uniform Nanostructured EC-SERS Active Working Electrode for Rapid Detection of Uric Acid. *Sensors*, *20*(24), 7066. https://doi.org/10.3390/s20247066

Jones, T., Zhou, D., Liu, J., Parkin, I. P., & Lee, T.-C. (2024). Quantitative multiplexing of uric acid and creatinine using polydisperse plasmonic nanoparticles enabled by electrochemical-SERS and machine learning. *Journal of Materials Chemistry B*, *12*(41), 10563–10572. https://doi.org/10.1039/D4TB01552E

Moldovan, R., Milenko, K., Vereshchagina, E., Iacob, B.-C., Schneider, K., Farcău, C., & Bodoki, E. (2023). EC-SERS detection of thiabendazole in apple juice using activated screen-printed electrodes. *Food Chemistry*, *405*, 134713. https://doi.org/10.1016/j.foodchem.2022.134713

Negri, P., Flaherty, R. J., Dada, O. O., & Schultz, Z. D. (2014). Ultrasensitive online SERS detection of structural isomers separated by capillary zone electrophoresis. *Chem. Commun.*, *50*(21), 2707–2710. https://doi.org/10.1039/C3CC49030K

Negri, P., Jacobs, K. T., Dada, O. O., & Schultz, Z. D. (2013). Ultrasensitive Surface-Enhanced Raman Scattering Flow Detector Using Hydrodynamic Focusing. *Analytical Chemistry*, *85*(21), 10159–10166. https://doi.org/10.1021/ac401537k

Negri, P., & Schultz, Z. D. (2014). Online SERS detection of the 20 proteinogenic <scp>l</scp> -amino acids separated by capillary zone electrophoresis. *The Analyst*, *139*(22), 5989–5998. https://doi.org/10.1039/C4AN01177E

Zhao, L., Blackburn, J., & Brosseau, C. L. (2015). Quantitative Detection of Uric Acid by Electrochemical-Surface Enhanced Raman Spectroscopy Using a Multilayered Au/Ag Substrate. *Analytical Chemistry*, *87*(1), 441–447. https://doi.org/10.1021/ac503967s
